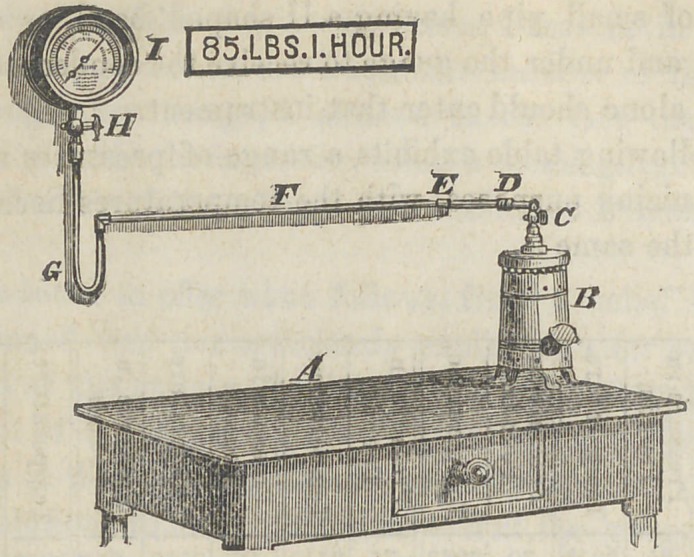# The Steam Guage in Vulcanizing

**Published:** 1865

**Authors:** A. Lawrence


### THE STEAM GUAGE IN VULCANIZING.

BY A. LAWRENCE.

In a short article of mine on Steam Pressure in Vulcanizers, which appeared in the December number of this Journal, among other things, I alluded to the fact that I had dispensed with the thermometer, using a steam guage instead; and stated some of the advantages secured, as I think, by the change.

I am induced to offer what follows, from a belief that the article named was not sufficiently explicite so far as relates to the *use* of the guage; this impression being strengthed by the receipt of letters of inquiry from several members of the profession in different parts of the country.

That solutions to such queries as "Will the guage fit vulcanizers of different manufactures?" "How do you ascertain the temperature in degrees?" "What does it look like," and "what is the price," &c., &c., are invited, does not surprise or annoy me in the least, for dentists, as a general thing, have had nothing to do with such an instrument, nor, until vulcanite came into use, with a thermometer even, except as the indicator of calorific fluctuations.

Without further regard to "Robin Hood's burn," permit me to say to all interested, that the guage most suitable for the purpose in question, somewhat resembles a small circular clock; is about six inches in diameter and marked to register one hundred and forty or one hundred and eighty pounds pressure, with pound dots near the outer circle of the dial. A pointer indicates the force which moves it.

This size is better than a smaller one, because the spring inside not being crowded to its utmost capacity in vulcanizing, will, of course, retain its working integrity longer; in fact, as long as any dentist now living will be personally interested in the matter. The price of such a guage, at this time, is $18.00; and, though more expensive ones can unquestionably be made, they are no more reliable, the difference consisting in mere "outward show and adorning." They can be used with all vulcanizers generating steam, connecting by means of three or four feet, or as much more as may be convenient, of small pipe having a U shaped bend, or a single coil near and under the guage to receive the condensed steam, as water alone should enter that instrument.

The following table exhibits a range of pressures sufficient for vulcanizing purposes, with the temperatures necessary to produce the same:

| Pressure in Pounds. | Temperature | Pressure in Pounds. | Temperature. | Pressure in Pounds. | Temperature. | Pressure in Pounds. | Temperature. | Pressure in Pounds. | Temperature. | Pressure in Pounds. | Temperature. | Pressure in Pounds. | Temperature. |
|---|---|---|---|---|---|---|---|---|---|---|---|---|---|
| 60 | 295° | 65 | 301° | 70 | 306° | 75 | 311° | 80 | 315° | 85 | 320° | 110 | 339° |
| 61 | 296° | 66 | 302° | 71 | 307° | 76 | 312° | 81 | 316° | 90 | 324° | 115 | 342° |
| 62 | 298° | 67 | 303° | 72 | 308° | 77 | 313° | 82 | 317° | 95 | 328° | 120 | 345° |
| 63 | 299° | 68 | 304° | 73 | 309° | 78 | 314° | 83 | 318° | 100 | 332° | 125 | 349° |
| 64 | 300° | 69 | 305° | 74 | 310° | 79 | 314° | 84 | 319° | 105 | 335° | 130 | 352° |

It will readily be seen by the above, that a pressure of sixty pounds requires a temperature of two hundred and ninety degrees by Fahrenheit's scale to produce it, and eighty-five pounds three hundred and twenty degrees, at which latter pressure I vulcanize, running one hour, and with the most satisfactory results.

The manner of putting up and using the guage is very simple. All that is required is to secure it, by screws passing through the flange on the back, in some conspicuous and convenient place, attach a pipe and carry it down ten or twelve inches, give it a bend or curve upward about half its length, or five or six inches, thence at right angels or otherwise, and in any convenient length not less than three feet, to the vulcanizer.

The annexed cut is from a photograph of a Whitney vulcanizer with the guage attached, but is by no means the only arrangement which can be made, as, in some cases, convenience may require more pipe, or a different distribution.

A Table or work bench. B Vulcanizer. C Side outlet pendant cock screwed on in place of the thermometer scale. D Coupling joint. E Angle in the pipe. F Iron pipe three-sixteenths inside. G U shaped curve five or six inches in depth. H Cock to the guage. I Guage.

The fitting, putting up and arranging the entire "fixins" can be done in an hours time by any gas fitter, or, to those residing away from cities or towns where such mechanics are employed, can be furnished to order by them, or by the parties furnishing the guage.

All the joints, from the vulcanizer to the guage, except the coupling, should be "leaded" with very thick lead paint, and screwed together steam tight.

In using the apparatus, the cocks C and H must be turned straight with the pipe, for if shut off at either point, the guage can not be acted upon by the steam. I generally heat the water in the vulcanizer nearly or quite to the boiling point, and let off the heated air by turning, or allowing to remain open, the cock C, then connect at the coupling D, turning the nut tight (not too tight) with a wrench.

So soon as steam begins to form, it is condensed by contact with the cold part of the pipe, and falls into and fills the curve or coil with water which is then forced into the guage with a power indicated by the pointer on the dial. The pipe should descend a trifle from the angle E to the commencement of the curve, to facilitate the passage of the condensed steam to that point.

Although vulcanizing one hour at eighty-five pounds affords results satisfactory to me, others may prefer a different time with more or less heat.

The table will be found a guide in such cases.

When the time is up, discontinue the fire, and shut off the steam by turning the cock C. Turn the cock H in the same manner, to prevent a too sudden reverse movement of the machinery of the guage, the pressure on which should be gradually relieved at any convenient time.

Now disconnect by unscrewing the coupling and dispose of the steam in the vulcanizer by blowing off, or any other means preferred. Further remarks would seem unnecessary to a full understanding of the subject. Having used the guage almost every day for about six months, I am fully satisfied that it is a decided improvement in vulcanizing, and am so delighted with it that no reasonable sum would induce me to substitute the thermometer.

Before closing, I may be permitted, in justice to the American Steam Guage Co., of which Mr. H. K. Moore is Superintendent, to correct an error in my former communication in locating their office at No. 44 Exchange, instead of No. 44 Congress street, Boston.

## Figures and Tables

**Figure f1:**